# A distinctive profile of family genetic risk scores in a Swedish national sample of cases of fibromyalgia, irritable bowel syndrome, and chronic fatigue syndrome compared to rheumatoid arthritis and major depression

**DOI:** 10.1017/S0033291722000526

**Published:** 2023-07

**Authors:** Kenneth S. Kendler, Judith G.M. Rosmalen, Henrik Ohlsson, Jan Sundquist, Kristina Sundquist

**Affiliations:** 1 Virginia Institute for Psychiatric and Behavioral Genetics and Department of Psychiatry, Virginia Commonwealth University, Box 980126, Richmond, VA 23298-0126, USA; 2 Departments of Psychiatry and Internal Medicine, University of Groningen, University Medical Center Groningen, P.O. Box 30.001, 9700 RB Groningen, Netherlands; 3 Center for Primary Health Care Research, Department of Clinical Sciences, Lund University Clinical Research Centre (CRC), Box 50332, SE-202 13 Malmö, Sweden

**Keywords:** Chronic fatigue syndrome, fibromyalgia, irritable bowel syndrome, major depression, rheumatoid arthritis

## Abstract

**Background:**

Functional somatic disorders (FSD) feature medical symptoms of unclear etiology. Attempts to clarify their origin have been hampered by a lack of rigorous research designs. We sought to clarify the etiology of the FSD by examining the genetic risk patterns for FSD and other related disorders.

**Methods:**

This study was performed in 5 829 186 individuals from Swedish national registers. We quantified familial genetic risk for FSD, internalizing disorders, and somatic disorders in cases of chronic fatigue syndrome (CFS), fibromyalgia (FM), and irritable bowel syndrome (IBS), using a novel method based on aggregate risk in first to fifth degree relatives, adjusting for cohabitation. We compared these profiles with those of a prototypic internalizing psychiatric – major depression (MD) – and a somatic/autoimmune disorder: rheumatoid arthritis (RA).

**Results:**

Patients with FM carry substantial genetic risks not only for FM, but also for pain syndromes and internalizing, autoimmune and sleep disorders. The genetic risk profiles for IBS and CFS are also widely distributed although with lower average risks. By contrast, genetic risk profiles of MD and RA are much more restricted to related conditions.

**Conclusion:**

Patients with FM have a relatively unique family genetic risk score profile with elevated genetic risk across a range of disorders that differs markedly from the profiles of a classic autoimmune disorder (RA) and internalizing disorder (MD). A similar less marked pattern of genetic risks was seen for IBS and CFS. FSD arise from a distinctive pattern of genetic liability for a diversity of psychiatric, autoimmune, pain, sleep, and functional somatic disorders.

Functional somatic disorders (FSD) are characterized by clusters of somatic symptoms of unknown origin which, in the absence of detectable pathological abnormalities, are diagnosed solely by symptoms (see Table 1 for a listing of all the abbreviations used in this manuscript). The three best-studied FSD are fibromyalgia (FM), irritable bowel syndrome (IBS), and chronic fatigue syndrome (CFS). Compared to chronic classical diseases with similar symptoms, FSD are associated with comparable reductions in quality of life and functioning (Joustra, Janssens, Bultmann, & Rosmalen, 2015). In many medical specialties, FSD outnumber more established chronic somatic diseases (Nimnuan, Rabe-Hesketh, Wessely, & Hotopf, 2001).

**Table 1.** Abbreviations used in this manuscript, listed alphabetically

Abbreviation	Meaning
AD	Anxiety disorder
BP	Back pain
CFS	Chronic fatigue syndrome
FGRS	Familial genetic risk score
FM	Fibromyalgia
FSD	Functional somatic disorders
GRA	Graves' disorder
HASH	Hashimoto's thyroiditis
IBD	Inflammatory bowel disorder
IBS	Irritable bowel syndrome
MD	Major depression
MIG	Migraine
PoRh	Polymyalgia rheumatica
RA	Rheumatoid arthritis
SD	Sleep disorder

To identify new treatment targets, many studies have tried to clarify the etiological mechanisms of FSD, identifying both shared and FSD-specific factors, including alterations in pain processing pathways, local and systemic immune activation (Andrés-Rodríguez et al., 2020; Burns et al., 2019; Strawbridge, Sartor, Scott, & Cleare, 2019), and the hypothalamic-pituitary-adrenal axis (Tak et al., 2011). However, most studies are cross-sectional so differences (e.g. in sleep or activity patterns or diet) could reflect causes of FSD or their consequences. Furthermore, candidate mechanisms are typically studied in isolation, revealing only modest case-control differences. New approaches to identify etiological mechanisms are urgently needed.

Clarification of the genetic background of FSD could provide insight into etiological mechanisms without the problem of reverse causality and facilitate the study of multiple pathways to illness. Several studies suggest a familial/genetic predisposition for FSD. Moderate levels of heritability were found in twin studies of FM (Dutta et al., 2020), IBS (Svedberg, Johansson, Wallander, & Pedersen, 2008), and CFS (Buchwald et al., 2001). Which particular genes are responsible remains unknown as genome-wide association studies of FSD are in early stages, with limited power and/or shallow phenotyping (Bonfiglio et al., 2018; Docampo et al., 2014; Schlauch et al., 2016). A recent large study identified six genetic susceptibility loci for IBS, of which four were associated with mood and anxiety disorders, expressed in the nervous system, or both (Eijsbouts et al., 2021).

Further insight into the nature of genetic vulnerabilities can be obtained by studying genetic liability shared with other disorders. Two relevant studies have been published in a large sample of female older adult Swedish twins. Wojczynski et al. performed a cotwin-control study of MD and IBS and suggested little to no genetic relationship between the two syndromes (Wojczynski, North, Pedersen, & Sullivan, 2007). By contrast, Kato et al. performed a multivariate twin study suggesting genetic links between internalizing psychiatric disorders and both IBS and CFS (Kato, Sullivan, Evengård, & Pedersen, 2009). Studies on shared genetic liability of FSD and somatic disorders are lacking, possibly due to the presumed psychological origin of FSD and the lack of high-quality data in samples of sufficient size.

An alternative approach to explore the familial/genetic background of FSD is through pedigree data which we utilize here. The recently developed family genetic risk score (FGRS) (Kendler et al., In press b; Kendler, Ohlsson, Sundquist, & Sundquist, 2021a, 2021b) is based on the aggregate risk for disorders in first to fifth degree relatives correcting for their age, sex, year of birth, and place of residence. Their data are weighted by their genetic relationship to the proband, and cohabitation effects are accounted for in parents and siblings to ‘subtract’ out the effects of familial-environmental factors. The FGRS is a phenotype-based measure of aggregate genetic risk and thus entirely different from genotype-based measures such as polygenic risk scores.

The aim of this study, therefore, is to quantify familial genetic risk, as assessed by the FGRS, for FSD, internalizing disorders, and a series of relevant somatic disorders in a population-based sample of individuals diagnosed with CFS, FM, and IBS ascertained from national Swedish Medical Registers. We then compare these profiles with those found for a prototypic internalizing and somatic disorder ascertained from the same source: major depression (MD) and rheumatoid arthritis (RA).

## Methods

We collected information on individuals from Swedish population-based registers with national coverage linking each person's unique personal identification number which, for confidentiality, was replaced with a serial number by Statistics Sweden. This study was approved by the Regional Ethical Review Board of Lund (No. 2008/409, 2012/795, and 2016/679).

Our database consisted of all individuals born in Sweden between 1932 and 1995 of Swedish born parents and followed through 31 December 2017. In the database, we included date of first registration for three FSD: FM, IBS, and CFS utilizing ICD-10 codes from Swedish national primary care, specialist, and hospital registries (Appendix Table 1), as well as dates of first registrations for MD and RA. To compare the genetic profiles, we also included data on eight disorders chosen for their potential relationship with one or more of the FSD and a sufficient prevalence to provide adequate power: anxiety disorders (AD), inflammatory bowel disease (IBD), Graves' disease (GRA), Hashimoto's thyroiditis (HASH), polymyalgia rheumatica (PoRh), back pain (BP), migraine (MIG), and sleep disorder (SD) (see Appendix Table 1 for ICD codes). Pain disorders (BP, MIG) and autoimmune disorders (GRA, HASH, RoPh) were included because of the presumed role of pain sensitivity (Lewis, Rice, & McNair, 2012) and autoimmunity (Goebel et al., 2021; Koloski et al., 2019; Sotzny et al., 2018) in the etiology of FSD. Sleep difficulties and disorders are known comorbidities in IBS (Shiha & Aziz, 2021), FM (Wu, Chang, Lee, Fang, & Tsai, 2017), and CFS (Jackson & Bruck, 2012). For MD and AD, we use ICD-8, ICD-9, and ICD-10 codes. For all other disorders, we utilize only ICD-10 codes. We divide these 10 disorders into four categories: *internalizing psychiatric disorders* (MD, AD); *autoimmune disorders* (IBD, GRA, RA, HASH, PoRh); *pain disorders* (BP, MIG); and *SD*.

Individual FGRSs were based on selected first through fifth degree relatives of the probands producing a mean of 40.1 relatives per proband. Briefly (see Appendix Table 2 for full details) for each disorder, we estimated the distribution of *age at first registration*. The empirical distribution was used to obtain weights for relatives without a registration for the disorder, to account for the proportion of the time-at-risk period they had completed at follow-up. Then we transformed the binary variable into a *Z*-score based on the threshold for each disorder and estimated the mean of the underlying liability to obtain sex and birth decade-specific *Z*-scores for relatives with the disorder and relatives without. For first degree relatives, we also calculated a factor to control for the shared environmental effect of cohabitation. For parent–offspring pairs, the factor was calculated from the resemblance of father–offspring pairs where the father sired and raised his child *v.* pairs where father sired their offspring but never lived with or near them when they grew up. For sibling pairs, we compared the resemblance in half-sibs who were *v.* were not reared together.

For each relative, we then had four components: the *Z*-score (reflecting sex and year of birth adjusted rates), weight (reflecting the proportion of risk period they had completed if unaffected), and proportion of shared genes with the proband. For each proband, we averaged the product of these four components. Finally, to obtain the individual FGRS, we multiplied the quotient with a shrinkage factor based on the variance of the *Z*-score across all relatives, the variance in the mean *Z*-score across all probands, and the weighted number of relatives for each proband. So that the FGRSs would be more comparable across disorders and to reduce the effect of register coverage, we standardized the FGRS by year of birth into a *Z*-score with mean = 0 and s.d. = 1. This *Z*-score can be best understood as the mean genetic risk of an individual compared to the Swedish general population.

To investigate the genetic profiles of FM, IBS, and CFS, we compared the mean value of the 13 FGRSs for individuals with FM and/or IBS and/or CFS. First, we performed an overall test of the mean values and thereafter the three unique comparisons (FM *v.* IBS, FM *v.* CFS, IBS *v.* CFS). The same approach was performed to compare the genetic profiles of FM, IBS, and CFS with profiles of MD and RA. All analyses were performed using SAS 9.4 (SAS Institute, 2012). Given the total of 117 non-independent tests performed, we used a conservative *p* value <0.0001 to determine significance.

## Results

### Descriptive statistics

Our cohort consisted of 5 829 186 individuals (Table 2) and contained 24 833 cases of FM (85.5% female), 186 740 cases of IBS (70.8% female), and 31 578 CFS (66.8% female). Appendix Fig. 1 details the modest overlap of cases of FM, IBS, and CFS.

**Table 2.** Population: individuals born in Sweden (1932–1995) to Swedish-born parents

	Total	Females	Males
*N*	5 829 186	2 846 494 (48.8%)	2 983 692 (51.2%)
Year of birth (mean, s.d.)	1963 (18.1)	1963 (18.1)	1963 (18.0)
Years of follow-up (mean, s.d.)	31.6 (13.7)	31.8 (13.8)	31.4 (13.6)
Fibromyalgia	24 833 (0.4%)	21 242 (0.8%)	3591 (0.1%)
Irritable bowel syndrome	186 740 (3.2%)	132 206 (4.6%)	54 534 (1.8%)
Chronic fatigue syndrome	31 578 (0.5%)	21 107 (0.7%)	10 471 (0.4%)
Rheumatoid arthritis	86 133 (1.5%)	58 347 (2.1%)	27 786 (0.95)
Major depression	704 963 (12.1%)	445 532 (15.7%)	259 431 (8.7%)

### Comparison of genetic profiles of FM, IBS, and CFS

**Fig. 1.**
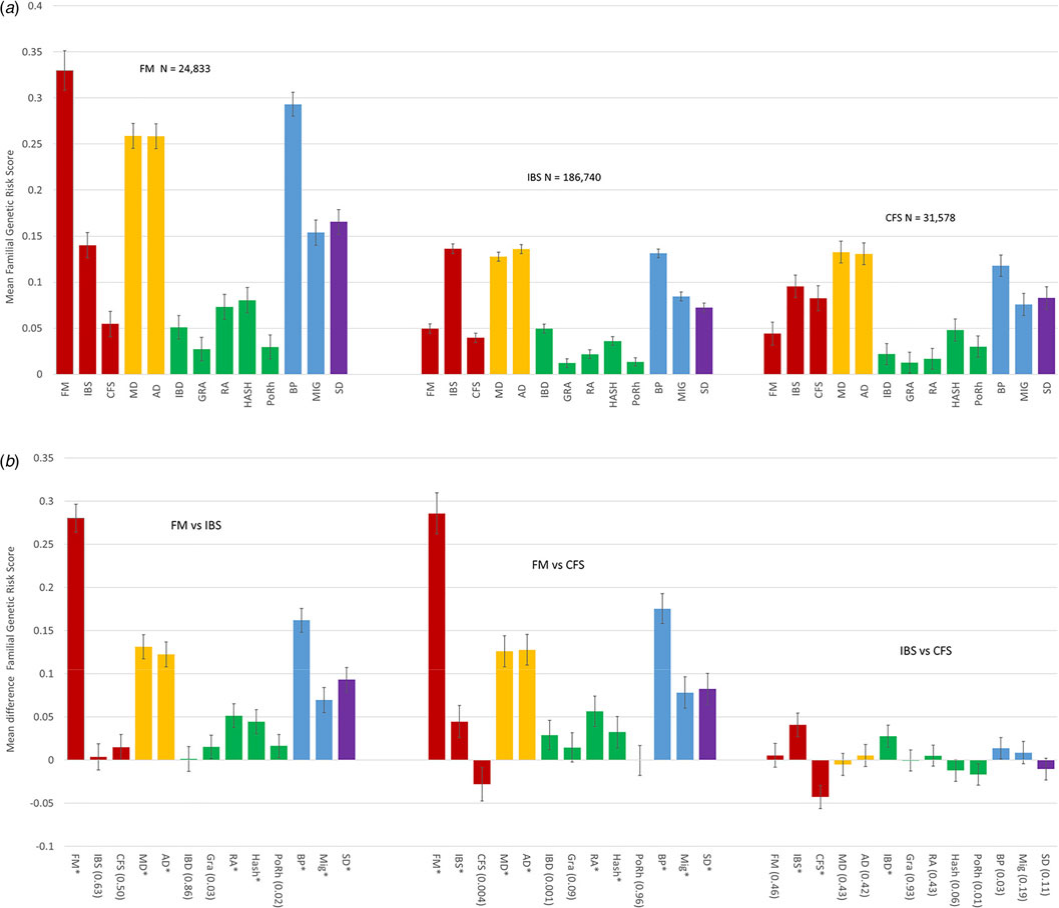
(*a*) The family genetic risk score (FGRS) profiles of individuals from the Swedish general population with diagnoses of fibromyalgia (FM), irritable bowel syndrome (IBS), and chronic fatigue syndrome (CFS). The sample sizes of these three groups are provided at the top of the figure. The mean FGRSs along with 95% confidence intervals are depicted on the *Y*-axis as a *Z*-score. The colors of the columns, for this and all subsequent figures, reflect the class of the disorders: red (black) – functional somatic disorders; yellow (dark grey) – internalizing disorders; green (grey) – autoimmune disorders; blue (light grey) – pain syndromes; purple (very light grey) – sleep disorders. The following initials are used for this and all subsequent figures: FM, fibromyalgia; IBS, irritable bowel syndrome; CFS, chronic fatigue syndrome; RA, rheumatoid arthritis; MD, major depression; IBD, inflammatory bowel disease; GRA, Graves' disease; HASH, Hashimoto's thyroiditis; PoRh, polymyalgia rheumatica; BP, back pain; MIG, migraine; SD, sleep disorders. (*b*) Mean differences in the family genetic risk score profiles (±95% CIs) between fibromyalgia (FM) and irritable bowel syndrome (IBS), fibromyalgia and chronic fatigue syndrome (CFS), and irritable bowel syndrome and chronic fatigue syndrome. The *Y*-axis depicts the mean differences in the FGRS *Z*-scores of the two disorders. A *p* value of the test of equality between mean values of the FGRS of <0.0001 is indicated by an asterisk (*). Otherwise the *p* value is placed in parentheses.

We present two figures comparing the FGRS profiles (with 95% CIs) for FM, IBS, and CFS. Figure 1*a* depicts, for patients with FM, IBS, and CFS, the FGRS for the three FSD and our 10 comparison disorders. Figure 1*b* presents the differences between the FGRS measures and their statistical significance (see Appendix Table 3 for tabular results).

Patients with FM have substantial genetic risks not only for FM, but also for IBS, internalizing psychiatric disorders, pain syndromes, several auto-immune disorders, especially RA and HASH, as well as SD (Fig. 1*a*). The genetic risk profiles for IBS and CFS suggest lower average risks but are also widely distributed across FSD, psychiatric, auto-immune, pain, and SD. The highest genetic risk for any autoimmune disorder in IBS patients is forIBD.

As seen in Fig. 1*b*, the differences in genetic risk profiles between FM and both IBS and CFS are more pronounced than between IBS and CFS. Compared to cases of IBS and CFS, patients with FM have substantially (and significantly) higher genetic risk for FM, MD, AD, RA, HASH, BP, MIG, and SD. Comparing IBS and CFS, we see only three significant differences. Each disorder has a higher primary FGRS (i.e. for the disorder itself) and IBS has a higher genetic risk to IBD.

### Comparison of genetic profiles of FM, IBS, and CFS with profiles of MD and RA

**Fig. 2.**
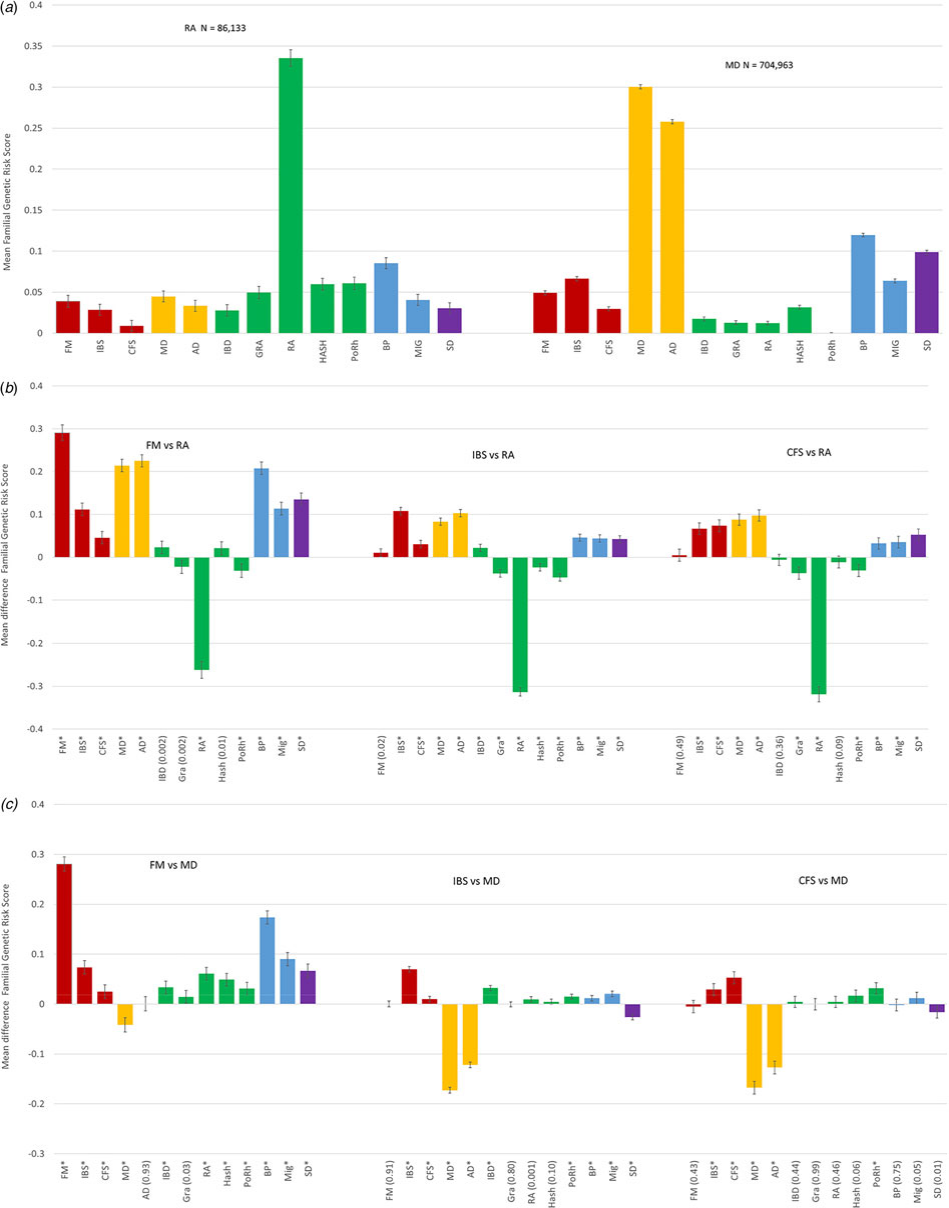
(*a*) The family genetic risk score profiles of individuals from the Swedish general population with diagnoses of rheumatoid arthritis (RA) and major depression (MD). The sample sizes of these two groups are provided at the top of the figure. The mean FGRSs along with 95% confidence intervals are depicted on the *Y*-axis as a *Z*-score. (*b*) Mean differences in the family genetic risk score profiles (±95% CIs) between fibromyalgia (FM) and rheumatoid arthritis (RA), irritable bowel syndrome (IBS) and rheumatoid arthritis, and chronic fatigue syndrome (CFS) and rheumatoid arthritis. The *Y*-axis depicts the mean differences in the FGRS *z*-scores of the two disorders. A *p* value of the test of equality between mean values of the FGRS of <0.0001 is indicated by an asterisk (*). Otherwise the *p* value is placed in parentheses. (*c*) Mean differences in the family genetic risk score profiles between fibromyalgia (FM) and major depression (MD), irritable bowel syndrome (IBS) and major depression, and chronic fatigue syndrome (CFS) and major depression. The *Y*-axis depicts the mean differences in the FGRS *Z*-scores of the two disorders. A *p* value of the test of equality between mean values of the FGRS of <0.0001 is indicated by an asterisk (*). Otherwise the *p* value is placed in parentheses.

To contextualize these findings, we compared the genetic profiles of our FSD with those of a classical autoimmune disorder – RA – and a classical psychiatric disorder: MD (Fig. 2*a*). The FGRS profile for RA is quite different from those of the FSD, characterized by one prominent elevated FGRS for RA itself and much more modest elevations for three of the four autoimmune disorders and for BP. By contrast, the FRGS profile for MD is more diverse, with substantial FGRS elevations for both MD and AD, with more modest elevated genetic risk for BP, SD, IBS, and MIG.

We then examined the statistical differences between the individual FGRS for FM, IBS, and CFS and first RA and then MD in, respectively, Fig. 2*b* and *c* (see Appendix Table 4 for results in tabular form). Compared to RA, FM had markedly lower genetic risk for RA and more modest but significantly lower risk for PoRh. FM had markedly higher genetic risk for FM, IBS, MD, AD, BP, MIG, and SD and more modest but significantly higher risk for CFS.

The differences between the genetic risk profile of RA and IBS and CFS were broadly similar with both disorders having markedly lower risk for RA and more modest reductions in risk for the other autoimmune disorders with one exception. IBS had a higher genetic risk for IBD than did RA. IBS and CFS had higher (and often significantly higher) risks for FSD, internalizing, pain, and SD.

The most striking result in Fig. 2*c* was the much lower genetic risk for MD and AD in IBS and CFS compared to MD patients. By contrast, for FM, the difference in genetic risk for MD was much more modest and absent for AD. The genetic risk for FM was much greater in the FM *v.* MD patients, and no such differences were seen for IBS and CFS. Compared to MD, genetic risk for BP, MIG, and SD were substantially higher only in the FM patients. While differences were more modest for the autoimmune disorders, all of them, especially RA and HASH, were clearly greater in the FM than MD patients. Smaller elevations for risk of several of the autoimmune disorders were seen in the IBS and FM *v.* MD patients, particularly elevated genetic risk for IBD in the IBS *v.* MD cases.

## Discussion

An examination of the pattern of disorders in extended pedigrees provides important information regarding the etiology of FSD. Patients with FM carry substantial genetic risks not only for FSD, but also for internalizing psychiatric disorders, pain syndromes, and autoimmune disorders and SD. The genetic risk profiles for IBS and CFS show lower average risks but are also similarly widely distributed across disorders.

By demonstrating significant genetic overlap with recognized somatic diseases, this study sheds new light on the long and controversial history of the nature of these disorders. Symptoms in the absence of objectifiable pathological abnormalities have classically been interpreted as resulting from psychological processes. In the absence of consistent pathological abnormalities, these syndromes have often been associated with the suspicion of psychoneurosis or malingering (Wessely & Hotopf, 1999). These biases have become barriers to etiological studies, reflected in low levels of funding and the lack of inclusion in most current large biobanks and cohort studies, with the few cohorts examining them utilizing only self-report measures. This absence of physician-rated phenotypic data in large samples has prevented the identification of etiological factors and consequently etiology-informed diagnostic criteria, resulting in a vicious circle due to the absence of a gold standard diagnosis for etiological studies. Current FSD diagnoses are based on polythetic diagnostic criteria including a wide range of symptoms, especially for FM and CFS, with no consensus on which criteria identify patients best, resulting in very heterogeneous patient profiles (Brurberg, Fønhus, Larun, Flottorp, & Malterud, 2014; Haney et al., 2015). Together with the possibility that each of these profiles can be the end product of multiple etiological pathways, this results in a heterogeneity that hampers the identification of etiological factors. Progress in this field urgently needs new empirical methods.

We have used such a new approach, and its results suggest that the etiology of FM, and to a somewhat lesser degree IBS and CFS, involves mechanisms related to pain sensitivity, depression, anxiety, and autoimmunity. We are the first to use a comprehensive analysis including multiple FSD and recognized somatic diseases, but our results fit earlier studies that showed shared genetic liability among IBS, chronic widespread pain and MIG (Vehof, Zavos, Lachance, Hammond, & Williams, 2014), and mood disorders (Bengtson, Aamodt, Vatn, & Harris, 2015; Kato et al., 2009). None of the previous studies of FSD examined genetic relationships with more traditional somatic disorders, although such associations would be informative for etiological pathways and could inform new treatment targets.

Our results are consistent with candidate etiological mechanisms including abnormalities in pain processing pathways in the central nervous system [FM (O'Brien, Deitos, Pego, Fregni, & Carrillo-de-la-Peña, 2018); FM, IBS, CFS (Lewis et al., 2012)] and in immune activation (Andrés-Rodríguez et al., 2020; Strawbridge et al., 2019). They also fit earlier genome-wide association study results suggesting a role for the central nervous system in FM (Docampo et al., 2014) as well as a recent study showing that mice treated with immunoglobulin G from FM patients developed FM-like symptoms (Goebel et al., 2021). Our results might also point to neuroinflammation as a shared mechanism. For example, activated microglia have been postulated to underpin comorbidity between CFS and depression (Chaves-Filho, Macedo, de Lucena, & Maes, 2019). The association between MD and autoimmune disorders is much more modest, arguing against a prominent role for immunological mechanisms in MD. The genetic profiles of the FSD are quite distinct from those of MD and RA, as, respectively, prototypic psychiatric and somatic diseases. Especially, elevated FGRSs in patients with RA are much more restricted to related chronic somatic diseases. The FRGS profile for MD is somewhat more diverse, with substantial elevations for both MD and AD, consistent with many prior studies suggesting these two disorders are closely genetically related (Kendler, Gardner, Gatz, & Pedersen, 2006).

While the FSD share a wide variety of genetic risks, our analyses provide novel insights into differences between these disorders. Based on their co-occurrence, overlap in diagnostic criteria, and shared risk factors, FSD have been viewed as largely an artefact of medical specialization (Wessely, Nimnuan, & Sharpe, 1999). Our results argue against this lumping approach, as the genetic risk profile for FM differs substantially from those seen for CFS and IBS. Notably, the genetic risks for internalizing disorders in patients with FM are very similar to those seen for MD, a pattern quite different from that observed for IBS and CFS. While the differences between CFS and IBS were subtler, they were meaningful. Each had significantly higher risk for their own FGRS and the FGRS for IBD was significantly stronger in IBS cases. Our results suggest at least some unique genetic pathways to each FSD, with an overall more prominent profile of shared genetic risks for FM than CFS or IBS. It is of interest to compare our findings with a prior multivariate twin study in older women (Kato et al., 2009). This study showed, consistent with our own results, shared genetic effects between MD, AD, and headache (which might be considered a ‘pain disorder’) and our three FSD: IBS, CFS, and FM. In addition, also broadly congruent with our findings, they showed an independent set of genetic factors influencing just the three FSDs as well as headache.

The results on FSD fit the multifactorial nature assumed to underlie these disorders. Our results cannot easily distinguish between a multifactorial etiology in an individual *v.* in the population. To explore this issue, we used latent class analysis (LCA) for FM to see if we could detect clear etiologic heterogeneity. We applied LCA to the FGRS for FM and for two of each group of associated disorders. Fit statistics suggested a three-class solution (Appendix Table 5), with the resulting solutions demonstrating quantitative rather than qualitative differences (Appendix Fig. 2). We found no support for the existence of subtypes of FM related to genetic risk for internalizing disorders, pain syndromes, or auto-immune disorders. So, a model for FM where most affected individuals carry genetic liability to these three syndromes (internalizing disorders, pain syndromes, or auto-immune disorder) appears to be the most plausible one.

The major strength of this study is the large dataset, with physician-based diagnoses of FSD and other chronic somatic and psychiatric diseases. Our conclusions are thus limited to those disorders that have led to healthcare-seeking resulting in a diagnosis. Given the controversy surrounding these disorders, FSD might be underdiagnosed. We cannot determine the degree to which the FSD were diagnosed by Swedish physicians according to the official diagnostic criteria. However, compared to these clinician-based diagnoses, most previous large-scale studies relied on self-report which rarely meet diagnostic criteria (Walitt, Katz, Bergman, & Wolfe, 2016). The comorbidity between FSD in our database was rather modest. The validity of our diagnoses is supported by the substantial familial aggregation of FM, and lower levels for IBS and CFS, as well as the strong aggregation for MD and AD. The validity of our MD and AD diagnoses has been supported by its prevalence, sex ratio, risk factors, and familial aggregation patterns (Sundquist, Ohlsson, Sundquist, & Kendler, 2017).

Our FGRS is an estimate of genetic risk reflecting aggregation of disease in close and distant relatives and is quite different from the now popular molecular polygenic risk score. It has the advantage of being based on the phenotypic liability directly rather than an index of that liability through a set of single-nucleotide polymorphisms. However, our adjustments for cohabitation are approximate but only have small effects on the overall score (Kendler, Ohlsson, Sundquist, & Sundquist, In press a; Kendler et al., 2021a, 2021b). Our final genetic risk scores are not highly sensitive to the various corrections involved in their calculation, as their deletion produces results that correlate highly with those from the full model with similar predictive power (Kendler et al., In press a, 2021a, 2021b). For these analyses, we examined whether the FGRS for FM, IBS, CF, RA, and MD were relatively stable across birth cohorts (1932–1995) and geography (24 counties). In Appendix Table 6, we show that these effects accounted for a mean of 0.13% (s.d. 0.11) of population variance. Appendix Figs 3 and 4 examine mean FGRSs for our three FSD by county of residence and year of birth with only rare results that were nominally significant. We have validated the FGRS by comparing it to a recently proposed quantitative family-history score [LT-FH(30)], showing in Swedish samples that, when matched for the relatives examined, the two scores correlated +0.94 (0.02). Furthermore, we have tested the FGRS by simulation, showing that it performs as expected as do our corrections for cohabitation (Appendix Table 7, Figs 5–9).

Some comorbidity was observed between our three FSD – with the strongest seen between FM and CFS (Appendix Fig. 1). We examined the possible impact of this comorbidity on our findings by imposing a hierarchy between these two disorders based on the severity of the symptoms. We placed CFS higher on this hierarchy than FM because CFS requires 6 months of symptoms instead of 3 for FM, and CFS requires limitations in functioning while FM does not. However, only 854 cases in our sample had both an FM and CFS diagnosis. In Appendix Fig. 10, we show an FRGS profile of FM with and without the imposed hierarchy and the difference in scores between the two. Very little change was seen by subtracting the comorbid cases from FM suggesting that the observed comorbidity is impacting very modestly on our findings.

Our genetic design permitted us to examine causes instead of consequences of FSD. In theory, shared genetic risk might be explained by disorders such as SD and internalizing disorders being consequences of FSD. However, we repeated our analyses after eliminating all cases of FM from relatives, finding only very small reductions in all FGRSs, and no changes in overall patterns. The results on sleep shed new light on the directionality of the association between sleep and FM. Sleep disruption has long been assumed to be the result of the pain that characterizes FM, but more recently it was hypothesized that sleep dysfunction might also be a pathogenic stimulus of FM (Choy, 2015). Our analyses provide support for this hypothesis, given that SD aggregate in the families of patients with FM, even when family members with FM are excluded from the analyses.

Adequate ICD codes for many of our disorders of interest were only available in ICD-10, which are available in Sweden since 1997. This means that for some of the affected older relatives used to form our FGRS, they would only have the disorder if they received the diagnoses later in life. Otherwise, they would represent false-negative diagnoses. We have tried to account for this in the calculations of the FGRS by using birth cohort-specific thresholds and modification of time-at-risk period for the different birth cohorts.

Case-identification for all of our disorders was by physician diagnosis as recorded in Swedish Medical registers. This mightintroduce some bias in our results, such as sampling more severe cases than might be ascertained through a population survey. For the FSDs, we examined the proportions who were identified through primary care, specialist out-patient, and in-patient registries (see Appendix Table 8). For all three FSDs, the majority were seen only in primary care, but that group was larger for CFS (95%), intermediate for IBS (67%), and lowest for FM (60%).

In conclusion, we present a new approach to unravel the etiology of FSD which has produced robust empirical evidence that FSD arise from a distinctive pattern of genetic liabilities for a diversity of psychiatric, autoimmune, pain, sleep, and FSD, with this pattern being more striking for FM than for IBS and CFS. The genetic associations here observed can guide much needed further research into etiological mechanisms.

## Supplementary material

For supplementary material accompanying this paper visit https://doi.org/10.1017/S0033291722000526. click here to view supplementary material
