# Supplementary material for: A distinctive profile of family genetic risk scores in a Swedish national sample of cases of fibromyalgia, irritable bowel syndrome, and chronic fatigue syndrome compared to rheumatoid arthritis and major depression
Source: Psychol Med. 2022 Mar 31;53(9):3879–86. doi: 10.1017/S0033291722000526 (PMC10317803; doi:10.1017/S0033291722000526)
Supplement: Supplementary file 1 [file S0033291722000526sup001.docx]

**APPENDIX**

**Definition of Phenotypes and Registries Used**

Based on the following registers: The Swedish Hospital Discharge Register (coverage 1973-2017); Outpatient Care Register (national coverage 2001-2017); Primary Care Registry (Partly coverage from 1999-2017)

Table 1 – ICD Codes for Diagnoses

|  | **ICD-codes** |
| --- | --- |
| Fibromyalgia (FM) | ICD-10: M79.7 |
| Irritable Bowel Syndrome (IBS) | ICD-10: K58 |
| Chronic Fatigue Syndrome (CFS) | ICD-10: G93.3 |
| Major Depression (MD) | ICD-8: 296.2, 298.0, 300.4; ICD-9: 296.2, 296.4, 298.0, 300.4; ICD-10: F32, F33. |
| Rheumatoid Arthritis (RA) | ICD-10: M05, M06 |
| Anxiety Disorders | ICD-8: 300.0, 300.2 ; ICD-9: 300A, 300C; ICD-10: F40, F41 |
| Inflammatory Bowel Disease | ICD-10: K51, K50 |
| Grave’s Disease | ICD-10: E05.0, E05.5 |
| Hashimoto’s Thyroiditis | ICD-10: E03.5, E03.8, E03.9, E06.3 |
| Polymyalgia Rheumatica | ICD-10: M35.3 |
| Back Pain | ICD-10: M54 |
| Migraine | ICD-10: G43, G44.00, G44.01, G44.02, G44.03, G44.04 |
| Sleep Disorder | ICD-10: G47 |
|  |  |

**Description of Registers**

Multi-Generation Register

The Multi-Generation Register is a register made up of persons who have been registered in Sweden at some time since 1961 and those who were born in 1932 or later. These are called index persons. The register contains connections between index persons and their biological parents. There are about 11 million index persons in the register. The Multi-Generation Register is a part of the register system for Total Population Register, where information comes from the National Tax Board. Every year, a new version of the register is created, including new index persons who immigrated or were born during the year. Information from the Multi-Generation Register may be disclosed for research and statistical purposes. For more information, see Statistics Sweden, Background Facts, Population and Welfare Statistics 2017:2, Multi-generation register 2016. A description of contents and quality

National Patient Register

In the 1960's the National Board of Health and Welfare started to collect information regarding in-patients at public hospitals, the National Patient Register (NPR). Initially it contained information about all patients treated in psychiatric care and approximately 16 percent of patients in somatic care. The register at that time covered six of the 26 county councils in Sweden. In 1984, the Ministry of Health and Welfare together with the Federation of County Councils decided a mandatory participation for all county councils. From 1987, NPR includes all in-patient care in Sweden. Since 2001, the register also covers outpatient doctor visits including day surgery and psychiatric care from both private and public caregivers. For more information, see https://www.socialstyrelsen.se/en/statistics-and-data/registers/register-information/the-national-patient-register/

Primary Care Registry

We also used information from our new Primary Care Registry (PCR), a research dataset including individual-level information on clinical diagnoses from primary health care centers from the following 15 of the 21 Swedish counties: Blekinge (2009-2017), Värmland (2005-2015), Kalmar (2007-2016), Sörmland (1997-2017), Uppsala (2005-2015), Västernorrland (2008-2015), Norrbotten (2009-2016), Gävleborg (2010-2016), Halland (2007-2014), Jönköping (2008-2014), Kronoberg (2006-2016), Skåne (1998-2013), Östergötland (1997-2014), Stockholm (2003-2016), and Västergötland (2000-2013). In 2016, these counties included 87% of the Swedish population. For more information see Sundquist, J., Ohlsson, H., Sundquist, K. et al. Common adult psychiatric disorders in Swedish primary care where most mental health patients are treated. BMC Psychiatry 17, 235 (2017).

**-----------------------------------------------------------------------------------------------------------------------------------**

**Table 2 - Calculation of the Familial Genetic Risk Score (FGRS):**

The dataset for the calculations includes:

Column 1 = Identification number of the proband (Born 1932-1995)

Column 2 = Identification number of the relative (1^st^ to 5^th^ degree relatives)

Column 3 = Proportion of shared additive genetic effects (0.03125 to 0.50) with the proband

Column 4 = Year of Birth of relative

Column 5 = Sex of relative

Column 6 = Age at registration for the disorder

Column 7 = Age at end of follow-up (2017-12-31 or age at death, or age at emigration whichever came first)

**Step 1:** Using all unique relatives with a registration for the disorder of interest, we non-parametrically estimated the distribution of *Age at first registration (*i.e., the crude distribution of Age at first registration among individuals in the sample born 1932-1995*).* This distribution is used to obtain weights for relatives without a registration for the disorder, in order to account for the proportion of the time-at-risk period they had completed at the end of follow-up. For example, for relatives at age x at the end of follow-up, the weight corresponds to the proportion of individuals registered for the disorder that had a registration for that disorder at age x. For relatives born prior to 1958 we subtracted age at the end of follow-up with the following formula: 1958 - Year of birth of relative. This modification was done in order to control for registration effects (i.e, most registers in Sweden start in 1973 suggesting that relatives from early birth cohorts do not have the possibility to be registered at younger ages). Note that all relatives with the disorder are weighted one.

**Step 2:** Transform the binary variable (disorder yes/no) into a z-score based on the threshold for each disorder (i.e., the Z-score of the p^th^ percentile of the normal distribution with mean 0 and standard deviation one, where p is the rate of the binary variable). This was done for males and females separately within each decade of birth (i.e., one specific threshold for males born 1930-1939, on specific threshold for females in the same birth cohort, one specific threshold for males born 1940-149 etc). As the underlying liability for a unique individual is not assessable, we estimated the mean of the underlying liability to obtain Z-scores for relatives with the disorder registration and relatives without the disorder. This was done by first generating n random numbers from a N(0, 1) distribution and estimating the mean for individuals registered with the disorder (i.e., mean Z-score for observations above the threshold) and for individuals without a registration (i.e., mean Z-score for observations below the threshold). As an example males born 1940-1949 who were registered for FM were assigned a Z-score of 3.21 while males born 1940-1949 without a FM registration were assigned a Z-score of -0.001 and females born 1940-1949 who were registered for FM were assigned a Z-score of 2.88 while females born 1940-1949 without a FM registration were assigned a Z-score of -0.02..

**Step 3**: Correct for cohabitation effects. To estimate the cohabitation effect (i.e. “shared environment”), we created a database with all individuals in the Swedish population born in Sweden 1955-1990. We also included the number of years, during ages 0-15, that individuals resided in the same household as their biological father. We thereby were able to define two kinds of families i) “not-lived-with” father families (offspring never resided for more than 1 year in the same household or in the same community as their biological father); ii) “lived-with” father (offspring resided a minimum of 13 year in the same household as their biological father. This gave us the possibility to estimate the genetic transmission (father to offspring) in “not-lived-with” fathers (as there should be no environmental transmission as they never shared the same household) as well as the genetic + environmental transmission in “lived-with” father (as they share the same household and thereby contribute with both genetic and environmental transmission). We performed a logistic regression analysis with the binary disorder in offspring as outcome and the binary disorder in father, type of father, and their interaction as predictors. We used the interaction term as the difference of effect between genes only and genes + environment. The same approach was performed for half-siblings where we compared those who were reared together versus reared apart. The following interaction terms were used in the calculations for each of our thirteen main disorders:

|  | Parent/Children | Siblings |
| --- | --- | --- |
| Fibromyalgia (FM) | 0.60 | 0.72 |
| Irritable Bowel Syndrome (IBS) | 0.99 | 0.86 |
| Chronic Fatigue Syndrome (CFS) | 0.54 | 0.74 |
| Major Depression (MD) | 0.80 | 0.85 |
| Rheumatoid Arthritis (RA) | 0.86 | 0.75 |
| Anxiety Disorders | 0.87 | 0.81 |
| Inflammatory Bowel Disease | 0.99 | 0.81 |
| Grave’s Disease | 0.75 | 0.68 |
| Hashimoto’s Thyroiditis | 0.67 | 0.64 |
| Polymyalgia Rheumatica | 0.71 | 0.82 |
| Back Pain | 0.90 | 0.89 |
| Migraine | 0.82 | 0.79 |
| Sleep Disorder | 0.97 | 0.92 |
|  |  |  |

**Step 4:** Calculate the product for each relative using the four components:

1. Z-score (reflecting sex and year of birth adjusted rates)
2. Weight (reflecting the proportion of risk period they had completed)
3. Cohabitation effects
4. Proportion of shared genes (0.03125 to 0.50) identical by descent with the proband

**Step 5:** Average the product calculated in step 4 across all relatives to a proband

**Step 6**: Correct for the number of relatives. We multiplied the results from step 5 with a shrinkage factor (SF): B / (B+A/C). It produces more shrinkage if B and C are small and A is large.

1. the variance of the Z-score of the disorder across all relatives,
2. the variance in the mean Z-score across all probands,
3. the weighted number of relatives for each proband (sum of Column 3 across each proband).

**Step 7:** Correct for differences by year of birth and county. There are 21 counties in Sweden. For each proband we used the county they had resided in during the maximum number of years (measured from 1969 and onwards) We standardized the risk score by year of birth and county of the proband into a Z-score with mean 0 and SD 1. This was then used as the FGRS in the analyses.

--------------------------------------------------------------------------------------------------------------------------------------

Figure 1 – Venn Diagram of the Patterns of Comorbidity for FM, IBS and CFS


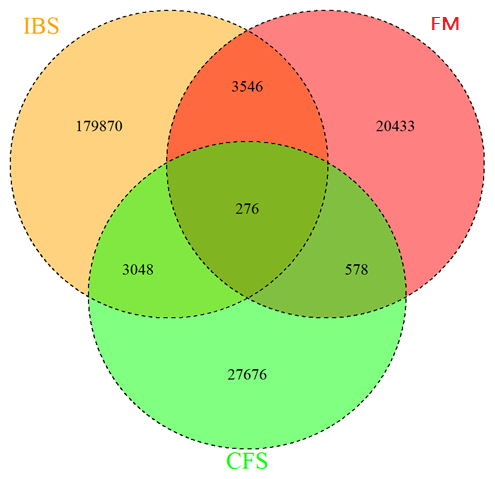


Table 3 – Statistical Comparison and Effect Size Difference of FGRS for Fibromyalgia, Irritable Bowel Syndrome and Chronic Fatigue Syndrome*

|  |  | Overall test | FM vs IBS | | FM vs CFS | | IBS vs CFS | |
| --- | --- | --- | --- | --- | --- | --- | --- | --- |
|  | *FGRS* |  | Difference | P-value | Difference | P-value | Difference | P-value |
| Fibromyalgia (FM) | **FM** | *<.0001* | 0.280 | *<.0001* | 0.286 | *<.0001* | 0.005 | 0.460 |
| Irritable Bowel Syndrome (IBS) | **IBS** | *<.0001* | 0.004 | 0.628 | 0.045 | *<.0001* | 0.041 | *<.0001* |
| Chronic Fatigue Syndrome (CFS) | **CFS** | *<.0001* | 0.015 | 0.050 | -0.028 | 0.004 | -0.043 | *<.0001* |
|  |  |  |  |  |  |  |  |  |
| Major Depression | **MD** | *<.0001* | 0.131 | *<.0001* | 0.126 | *<.0001* | -0.005 | 0.431 |
| Anxiety Disorders | **AD** | *<.0001* | 0.123 | *<.0001* | 0.128 | *<.0001* | 0.005 | 0.422 |
| Inflammatory Bowel Disease | **IBD** | *<.0001* | 0.001 | 0.860 | 0.029 | 0.001 | 0.028 | *<.0001* |
| Grave’s Disease | **GRA** | 0.090 | 0.015 | 0.029 | 0.015 | 0.093 | -0.001 | 0.928 |
| Rheumatoid Arthritis | **RA** | *<.0001* | 0.051 | *<.0001* | 0.056 | *<.0001* | 0.005 | 0.425 |
| Hashimoto’s Thyroiditis | **HASH** | *<.0001* | 0.044 | *<.0001* | 0.032 | *< .0001* | -0.012 | 0.061 |
| Polymyalgia Rheumatica | **PoRh** | 0.004 | 0.016 | 0.019 | 0.000 | 0.959 | -0.017 | 0.008 |
| Back Pain | **BP** | *<.0001* | 0.162 | *<.0001* | 0.175 | *<.0001* | 0.013 | 0.034 |
| Migraine | **MIG** | *<.0001* | 0.070 | *<.0001* | 0.078 | *<.0001* | 0.009 | 0.193 |
| Sleep Disorder | **SD** | *<.0001* | 0.019 | *<.0001* | 0.007 | *<.0001* | -0.012 | 0.106 |
| *P value <0.0001, depicted in italics, is considered significant. | | | | | | | | |

Table 4 Statistical Comparison of FGRS for Fibromyalgia, Irritable Bowel Syndrome and Chronic Fatigue Syndrome with Rheumatoid Arthritis and Major Depression

|  | FM vs RA | | IBS vs RA | | CFS vs RA | | FM vs MD | | IBS vs MD | | CFS vs MD | |
| --- | --- | --- | --- | --- | --- | --- | --- | --- | --- | --- | --- | --- |
|  | Difference | P-value | Difference | P-value | Difference | P-value | Difference | P-value | Difference | P-value | Difference | P-value |
| Fibromyalgia (FM) | 0.291 | *<.0001* | 0.010 | 0.023 | 0.005 | 0.492 | 0.281 | *<.0001* | 0.000 | 0.905 | -0.005 | 0.425 |
| Irritable Bowel Syndrome (IBS) | 0.111 | *<.0001* | 0.108 | *<.0001* | 0.067 | *<.0001* | 0.074 | *<.0001* | 0.070 | *<.0001* | 0.029 | *<.0001* |
| Chronic Fatigue Syndrome (CFS) | 0.046 | *<.0001* | 0.031 | *<.0001* | 0.073 | *<.0001* | 0.025 | *<.0001* | 0.010 | *<.0001* | 0.053 | *<.0001* |
|  |  |  |  |  |  |  |  |  |  |  |  |  |
| Major Depression | 0.214 | *<.0001* | 0.083 | *<.0001* | 0.088 | *<.0001* | -0.041 | *<.0001* | -0.173 | *<.0001* | -0.168 | *<.0001* |
| Anxiety Disorders | 0.225 | *<.0001* | 0.103 | *<.0001* | 0.097 | *<.0001* | 0.001 | 0.927 | -0.122 | *<.0001* | -0.127 | *<.0001* |
| Inflammatory Bowel Disease | 0.023 | 0.002 | 0.022 | *<.0001* | -0.006 | 0.364 | 0.033 | *<.0001* | 0.032 | *<.0001* | 0.004 | 0.444 |
| Grave’s Disease | -0.022 | 0.002 | -0.038 | *<.0001* | -0.037 | *<.0001* | 0.014 | 0.029 | -0.001 | 0.804 | 0.000 | 0.987 |
| Rheumatoid Arthritis | -0.262 | *<.0001* | -0.314 | *<.0001* | -0.319 | *<.0001* | 0.061 | *<.0001* | 0.010 | *0.001* | 0.004 | 0.464 |
| Hashimoto’s Thyroiditis | 0.021 | 0.005 | -0.024 | *<.0001* | -0.011 | 0.093 | 0.049 | *<.0001* | 0.004 | 0.101 | 0.017 | 0.006 |
| Polymyalgia Rheumatica | -0.031 | *<.0001* | -0.047 | *<.0001* | -0.031 | *<.0001* | 0.031 | *<.0001* | 0.015 | *<.0001* | 0.032 | *<.0001* |
| Back Pain | 0.208 | *<.0001* | 0.046 | *<.0001* | 0.032 | *<.0001* | 0.174 | *<.0001* | 0.012 | *<.0001* | -0.002 | 0.754 |
| Migraine | 0.113 | *<.0001* | 0.044 | *<.0001* | 0.035 | *<.0001* | 0.090 | *<.0001* | 0.021 | *<.0001* | 0.012 | 0.050 |
| Sleep Disorder | 0.135 | *<.0001* | 0.042 | *<.0001* | 0.053 | *<.0001* | 0.067 | *<.0001* | -0.026 | *<.0001* | -0.016 | 0.009 |
| P value <0.0001, depicted in italics, is considered significant. | | | | | | | | | | | | |

**Table 5 – Latent Class Analysis of FM - Fit of Various Classes: *Best-Fit Model**

| # Classes | AIC | Log-liklihood | Entropy |
| --- | --- | --- | --- |
| 2 | 219,227 | - 109,573 | 0.638 |
| 3 | 218,745 | - 109,311 | 0.501* |
| 4 | 218,557 | - 109,196 | 0.518 |
| 5 | 218,431 | - 109,111 | 0.509 |
| 6 | 218,403 | - 109,077 | 0.530 |

**Figure 2 Latent Class Analysis – 3 Class Solution**

**
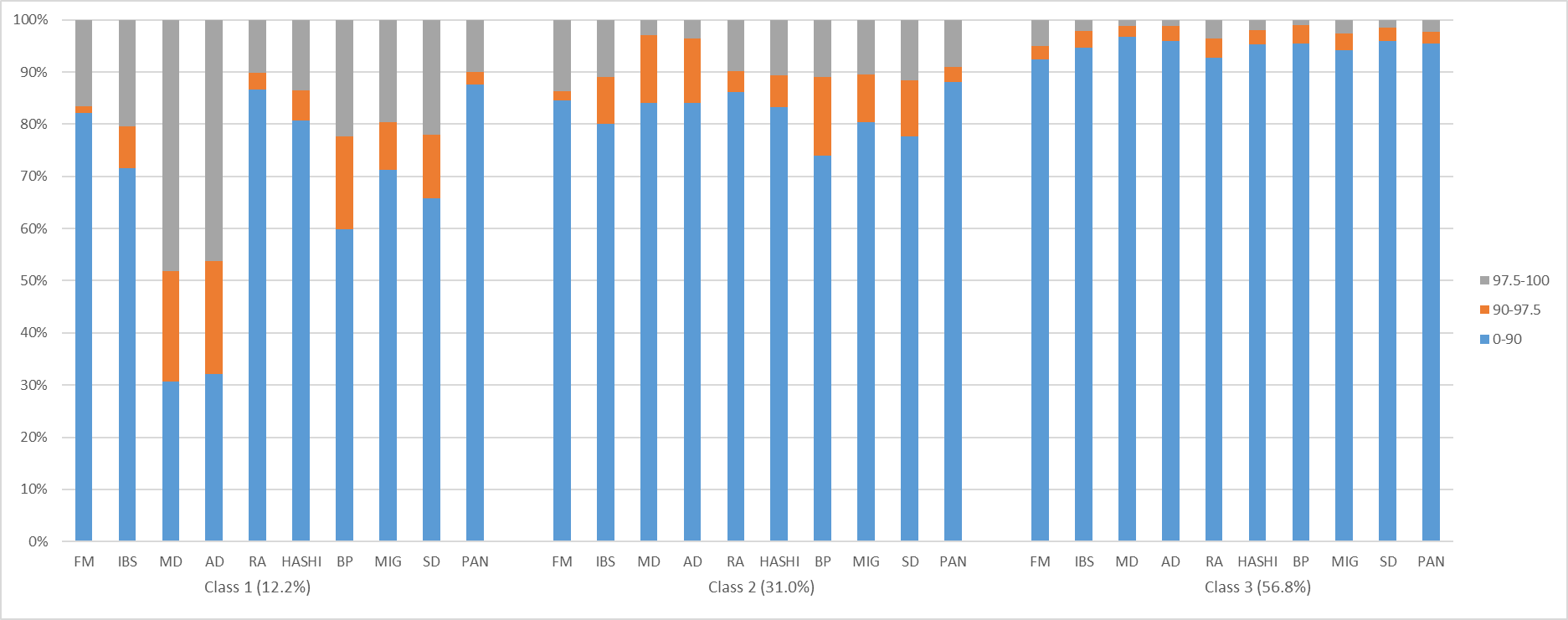
**

**Validity Checks on FGRS Scores for FM, IBS, CFS, RA and MD**

Table 6 - Stability (Intraclass Correlation) of FGRS Scores across Geography (24 counties) and Year of Birth (1932-1995).

|  | ICC_Geography_ | ICC_Year of birth_ |
| --- | --- | --- |
| FM | 0.4% | 0.1% |
| IBS | 0.01% | 0.01% |
| CFS | 0.1% | 0.1% |
| RA | 0.2% | 0.1% |
| MD | 0.1% | 0.2% |
|  |  |  |

Figure 3

Second level residual from a multilevel regression model with county as second level (Red dots = northern counties, and blue dots southern counties)


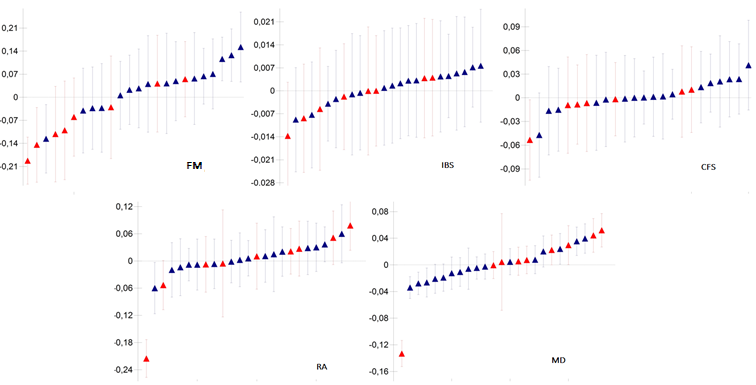


Figure 4 - Second level residual from a multilevel regression model with year of birth as second level (Red dots = northern counties, and blue dots southern counties)


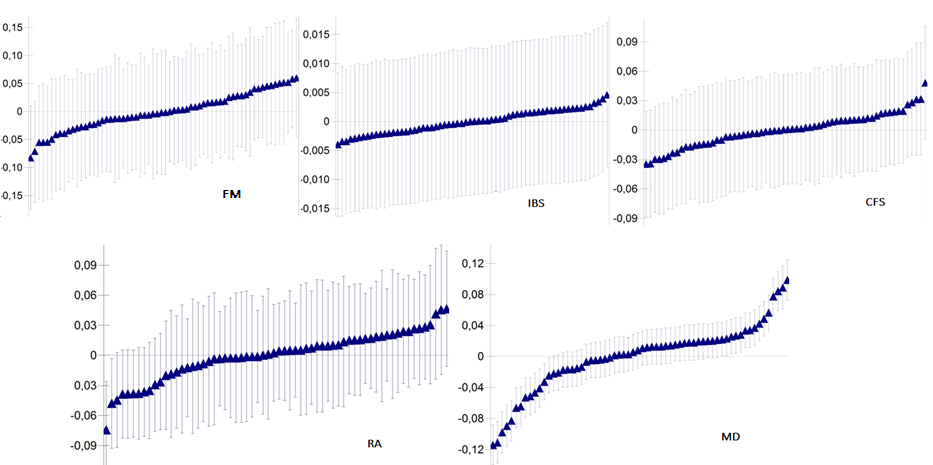


Table 7 – Simulation Methods

To get realistic (Swedish population-like) simulations, after unsuccessful attempts to utilize the *R* pedigree simulating packages *pedSimulate* and *synbreed,* we implemented a de-novo pedigree simulation using Julia script because of its greater speed. For increased generality, the script was built to have numerous adjustable parameters:

1. Additive heritability of the trait [setting for this manuscript (SFTM) h^2^= {20%, 40%, 60%, 80%}],
2. variance of siblings’ trait that is explained by the common or shared environment (SFTM c^2^= {2.5%, 5%, 10%, 20%}) which was applied only for full siblings,
3. k=number of generations (SFTM: k=5, i.e., founder generation gen=0 and gen=1- 4 for subsequent generations),
4. vector of average number of children per couple in generations 0 to k-2 (SFTM: $\mu=${2.1, 2.2, 1.7, 1.7}, as estimated from Swedish registries assuming average generation time is 25 years),
5. number of founders (SFTM: n=500K),
6. number of independent breeding groups (SFTM m=500, rather similar to villages),
7. (to avoid inbreeding) number of subgroups=k-1 for mothers in a subgroup to breed circularly with fathers from the next subgroup. (Children inherit the subgroup of mothers.)

The theoretical algorithm simulations were as follows:

1. Simulate independent True Breeding Values (TBV) for founder generation (gen=0), i.e. ${TBV}_{j}=\sqrt{h^{2}}* Z_{j}$, where $Z_{j}$ are independent standard normal (Gaussian) variates (j=1,…n),
2. For subsequent generations (gen=i>0)
   1. Within each group
      1. Permute mothers from one subgroups and fathers from the next,
      2. Pair mothers and fathers with the same rank,
      3. For each pair, simulate number of sibs m~ Poisson ($\mu_{i}$),
      4. If m>0, within each sibship
         1. Simulate sib’s j TBV_j_ as the sum of parent’ average and mendelian sampling, i.e., sib ${TBV}_{j}=\frac{{TBV}_{mother}+{TBV}_{father}}{2}+\sqrt{\frac{h^{2}}{2}} Z_{j}$, where $Z_{j}$ are independent standard normal (Gaussian) variates (j=1,…m),
         2. Simulate the common environment for all sibs within family as, $C= \sqrt{c^{2}} Z$, with $Z$ a single Gaussian variant for entire sibship,
         3. Simulate the independent environment for each sib within family as, $E_{j}= \sqrt{1-h^{2}-c^{2}} Z_{j}$, where $Z_{j}$ are independent standard normal (Gaussian) variates (j=1,…m),
         4. Compute liability for each sib as $L_{j}= {TBV}_{j}+C+E_{j}$
         5. Compute the affected status for each sib using the liability using a liability threshold model (for computational efficiency, computing affected status for multiple prevalences in a single pass).

Our simulations contained a mean (SD) of 324,656 (1,105) probands, each proband having a mean number of 3.7 (SD: 1.3) 1st degree relatives, 7.4 (SD: 1.8) 2nd degree relatives, 13.7 (SD: 4.3) 3rd degree relatives, and 23.3 (5.2) 4th degree relatives for a total mean number of relatives: 48.1 (SD:8.7).

Figure 5 Results of Simulations of Pedigrees Containing 1st-5th Degree Relatives Analyzed by FGRS as a Function of Heritability and Prevalence

For Figures 5-9 – see above for simulation methods


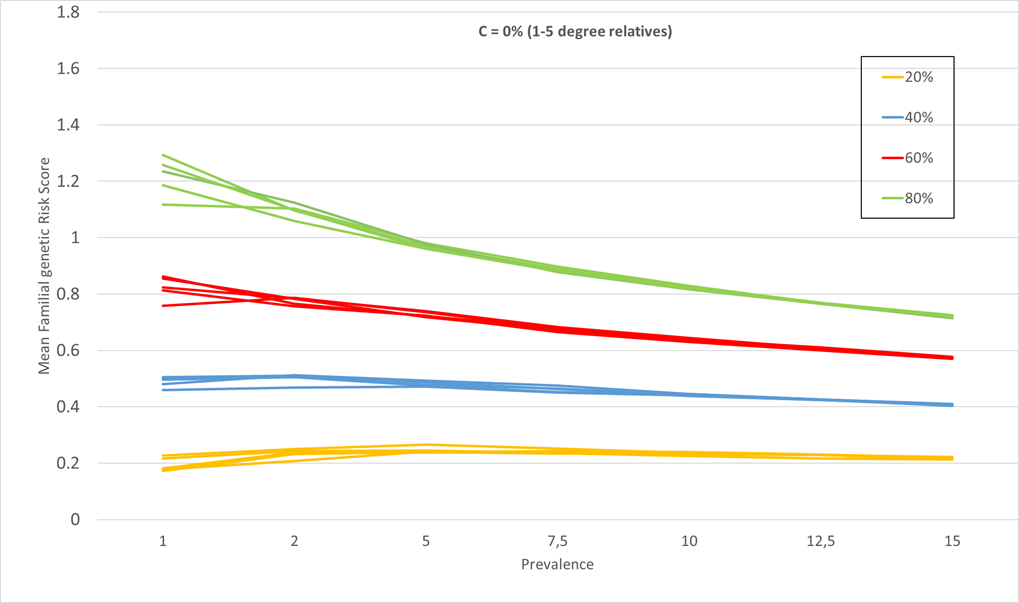


Figure 6

For Figures 6-8, we included in our simulations, estimates of shared environment for siblings with c2 equal to, respectively, 2.5, 5 and 10%. The thick colored lines are the estimates with the addition of the shared environment. The dotted line are those calculaed with the c2 parameter added. We then ”correct” for that sibling effect with 4 values of ”down-weighting”: 0.8, 0.6, 0.4, 0.2, which are represented by the thinner lines in the figures. The down-wighting values used in this paper are seen above in table 4 step 3.


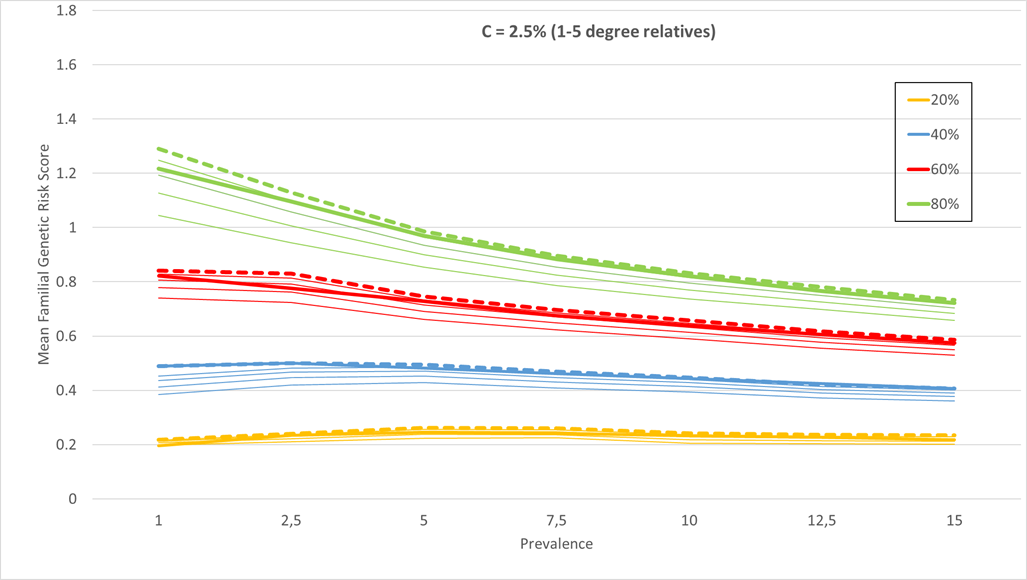


Figure 7


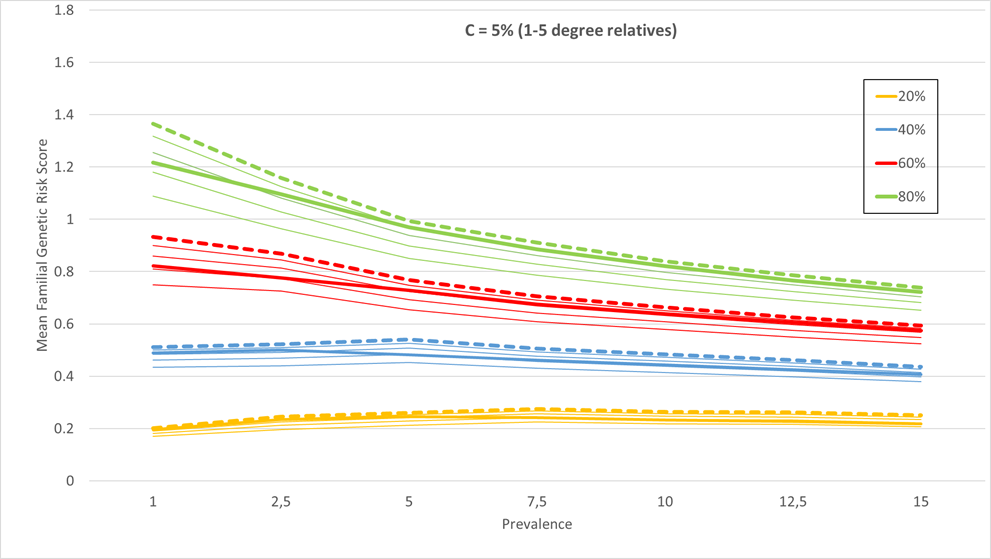


Figure 8


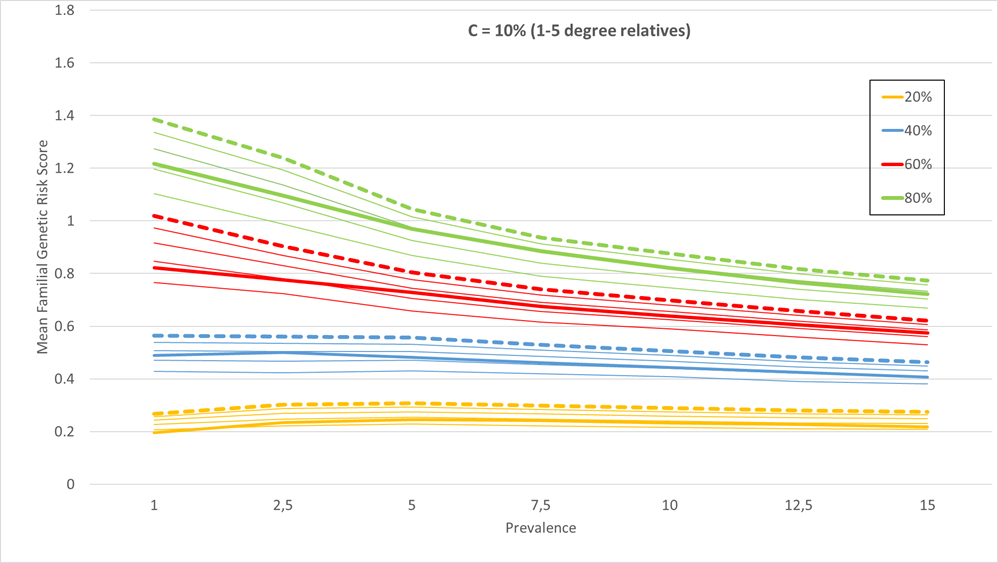


Figure 9

FGRS Profile for all Cases of FM (left), Cases of FM minus those comorbid with CF (center) and (right) the Difference in FGRS Profile between Them.


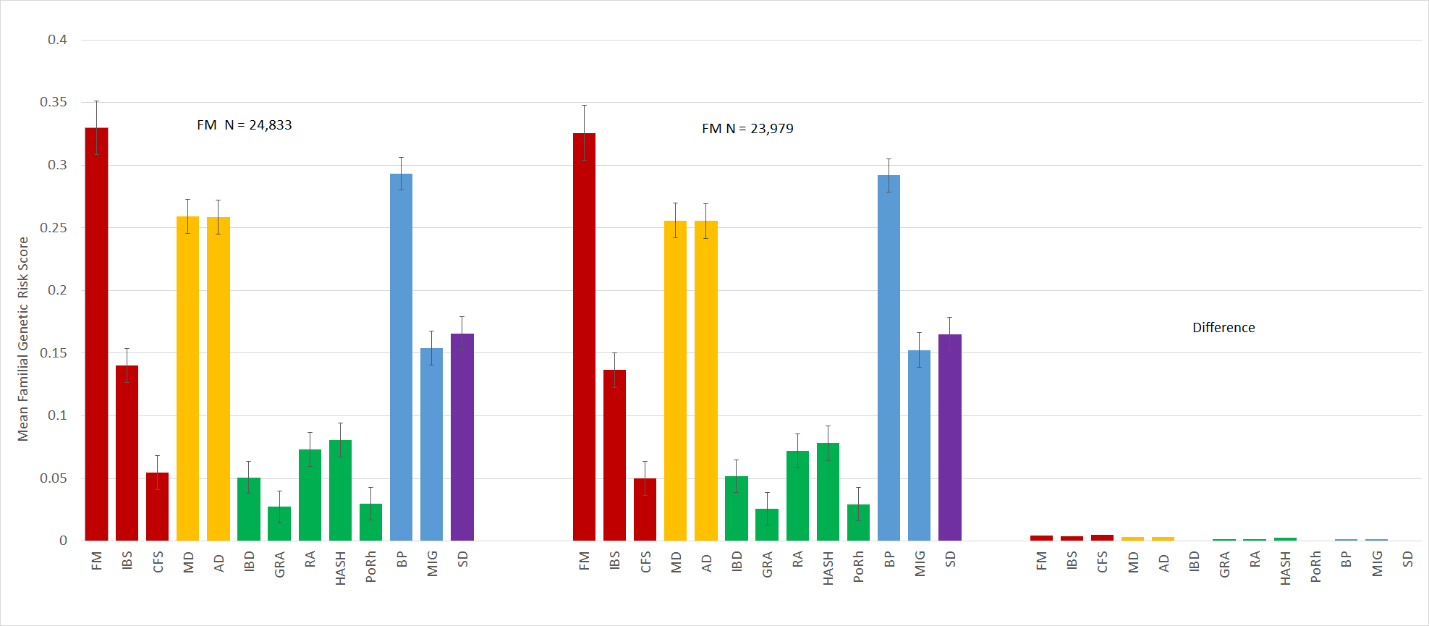


**Table 8 Registrations for FSDs**

(IP = Hospital (Inpatient register); OP = Specialist (Outpatient register); PC = Primary Care (Primary Care register)

|  | FM | IBS | CFS |
| --- | --- | --- | --- |
| IP + OP + PC | 0.1% | 0.4% | 0.0% |
| IP + OP | 0.4% | 0.4% | 0.1% |
| IP + PC | 0.7% | 0.3% | 0.1% |
| OP + PC | 7.1% | 12.7% | 1.2% |
| IP only | 15.8% | 1.6% | 0.6% |
| OP only | 16.4% | 17.8% | 2.9% |
| PC only | 59.5% | 66.8% | 95.1% |
|  |  |  |  |
